# Procalcitonin Levels in Gram-Positive, Gram-Negative, and Fungal Bloodstream Infections

**DOI:** 10.1155/2015/701480

**Published:** 2015-03-17

**Authors:** Christian Leli, Marta Ferranti, Amedeo Moretti, Zainab Salim Al Dhahab, Elio Cenci, Antonella Mencacci

**Affiliations:** Microbiology Section, Department of Experimental Medicine, University of Perugia, 06100 Perugia, Italy

## Abstract

Procalcitonin (PCT) can discriminate bacterial from viral systemic infections and true bacteremia from contaminated blood cultures. The aim of this study was to evaluate PCT diagnostic accuracy in discriminating Gram-positive, Gram-negative, and fungal bloodstream infections. A total of 1,949 samples from patients with suspected bloodstream infections were included in the study. Median PCT value in Gram-negative (13.8 ng/mL, interquartile range (IQR) 3.4–44.1) bacteremias was significantly higher than in Gram-positive (2.1 ng/mL, IQR 0.6–7.6) or fungal (0.5 ng/mL, IQR 0.4–1) infections (*P* < 0.0001). Receiver operating characteristic analysis showed an area under the curve (AUC) for PCT of 0.765 (95% CI 0.725–0.805, *P* < 0.0001) in discriminating Gram-negatives from Gram-positives at the best cut-off value of 10.8 ng/mL and an AUC of 0.944 (95% CI 0.919–0.969, *P* < 0.0001) in discriminating Gram-negatives from fungi at the best cut-off of 1.6 ng/mL. Additional results showed a significant difference in median PCT values between Enterobacteriaceae and nonfermentative Gram-negative bacteria (17.1 ng/mL, IQR 5.9–48.5 versus 3.5 ng/mL, IQR 0.8–21.5; *P* < 0.0001). This study suggests that PCT may be of value to distinguish Gram-negative from Gram-positive and fungal bloodstream infections. Nevertheless, its utility to predict different microorganisms needs to be assessed in further studies.

## 1. Introduction

Procalcitonin (PCT) is a 116-amino-acid peptide whose high levels are strongly associated with systemic bacterial infections [1, 2] and with the severity of illness [3]. It is produced in response to bacterial endotoxin and inflammatory host cytokines [4] and may help in discriminating bacterial from viral infections [4] and true bacteremia from contaminated blood cultures [5, 6]. It is known that Gram-positive or Gram-negative bacteria or fungi activate different Toll-like receptor (TLR) signaling pathways, resulting in production of different proinflammatory cytokines that ultimately stimulate PCT release [7]. This notion suggests that different pathogens could lead to different levels of PCT production. This issue could be of particular relevance in bloodstream infections, in which PCT could assist clinicians in setting the most appropriate early therapeutic approach that is essential for patient outcome [8]. Indeed, an inappropriate initial antimicrobial therapy is an independent risk factor for adverse outcome in patients with bloodstream infections from* Staphylococcus aureus* [9, 10] or Gram-negatives [11, 12].

Few studies are available in the literature on PCT utility in differentiating among Gram-negative, Gram-positive, or fungal bacteremias [13–15]. The aim of the present study was to evaluate PCT ability to discriminate different bacterial or fungal etiology in a large population of patients with documented bloodstream infection.

## 2. Materials and Methods

### 2.1. Patients and Samples

This prospective observational study was conducted using clinical and routine laboratory data collected from the Clinical Microbiology Unit of the General Hospital of Perugia, Italy, from March to September 2014.

Inclusion criteria were unselected consecutive blood samples for blood culture (BC) and PCT that, according to our hospital standard protocol, were collected simultaneously from each patient with suspected sepsis, defined according to Bone et al. [16]. Only patients older than 18 years of age were included in the study. For each patient, only one bloodstream infection episode and, for each episode, only the first samples were considered. In the case of two or more episodes observed in the same patient, only the first was considered. A bloodstream infectious episode was defined as a time-period associated with one or more blood cultures positive for the same organism/organisms [17, 18]. Exclusion criteria were lack of at least one of the above samples or samples not drawn simultaneously from the same patient.

### 2.2. PCT Determination

PCT levels were measured in sera via the automatic analyser VIDAS B.R.A.H.M.S. PCT assay (bioMérieux, Marcy l'Etoile, France), according to the manufacturer's instructions. The lower limit of detection of the assay was 0.05 ng/mL and the functional assay sensitivity was 0.09 ng/mL (VIDAS B.R.A.H.M.S. PCT package insert; bioMérieux).

### 2.3. Blood Culture

For each sample, an aliquot of 5 to 10 mL whole blood was inoculated into BACTEC aerobic and anaerobic bottles (Becton Dickinson, Sparks, MD). BACTEC Plus bottles were used for patients under antibiotic therapy and standard bottles for untreated patients. Two sets from two different sites were collected at the same time. The bottles were incubated in a BACTEC FX automated blood culture system (Becton Dickinson). All bottles flagged positive were removed from the instrument and an aliquot was taken for Gram-stain and culture on solid media for subsequent analysis. Identification of microorganisms was performed with conventional methods and with the matrix-assisted laser desorption/ionization-time-of-flight mass spectrometry (Bruker Daltonics, Bremen, Germany).

### 2.4. Definition of Pathogen

Microorganisms detected by BCs were considered to be clinically relevant pathogens rather than contaminants according to the following conditions: (i) microorganisms identified by two or more BCs, reported by the clinician as the cause of the episode of sepsis; (ii) microorganisms detected by only one set of BCs if coincided with the results of culture from samples from the suspected infectious foci, collected from the same patient during the same infectious episode; (iii) microorganisms detected only in one set of BCs, belonging to a species included among the etiopathogenic agents of the patient infectious disease (e.g.,* Streptococcus pneumoniae* from a patient with lobar pneumonia); (iv) microorganisms detected only in one set of BCs reported by the clinician as the cause of the episode of sepsis in the final diagnosis, based on clinical, instrumental, and laboratory data. Coagulase-negative staphylococci,* Corynebacterium* spp., and other skin commensals were considered contaminants when isolated from only one set of BCs [19] and in the absence of clinical and/or laboratory data suggesting their pathogenic role.

### 2.5. Statistical Analysis

Values were expressed as count and percentages or median and interquartile range (IQR). Statistical significance was assumed if a null hypothesis could be rejected at a *P* value of <0.05. The chi-square test was used to analyze associations between categorical variables. Multiple comparisons of continuous variables were assessed by the Kruskal–Wallis one-way analysis of variance. Receiver operating characteristics (ROC) curve analysis was used to define the diagnostic ability of the various PCT cut-offs, and Youden's indices were calculated to find the best discriminatory cut-off (Youden's index = sensitivity + specificity − 1). SPSS statistical package, release 13.0 (SPSS Inc., Chicago, IL), was used for all statistical analyses.

### 2.6. Ethic Statement

Samples were collected as part of standard care and those included in the database were deidentified before access. No personal information was stored in the study database. No patient intervention occurred with the obtained results. For these reasons, the study was exempt from the institutional review board.

## 3. Results

During the entire study period, a total of 8,752 BCs were collected from 3,651 patients. PCT was not drawn concomitantly with the first BCs in 1,702 patients that were excluded from the study. A total of 1,949 patients fulfilled the inclusion criteria and were enrolled in the study. Demographic characteristics of the patients and results from BCs are described in Table 1. Among 586 monomicrobial BCs, 345 (59%) were positive for Gram-negative, 217 (37%) for Gram-positive, and 24 (4%) for fungal pathogens.* Escherichia coli* (183 isolates, 31.2%) and* Staphylococcus aureus* (103 isolates, 17.6%) were the most frequent isolated organisms.

Antimicrobial therapy was already administered in 79.5% of patients with negative BCs, 74.6% with contaminated BCs, 82.6% with Gram-negative pathogens, 77.5% with Gram-positive pathogens, 87.5% with fungal pathogens, and 60% with polymicrobial sepsis. The rates of patients assuming antimicrobial therapy according to BC results showed no significant differences (*P* = 0.336).

PCT median value of positive BCs (6.72 ng/mL, IQR 1.5–23.3) was significantly higher than those observed in negative BCs (0.3 ng/mL, IQR 0.1–0.9, and *P* < 0.0001) or in contaminated BCs (0.2 ng/mL, IQR 0.1–0.5, and *P* < 0.0001).

**Figure 1:**
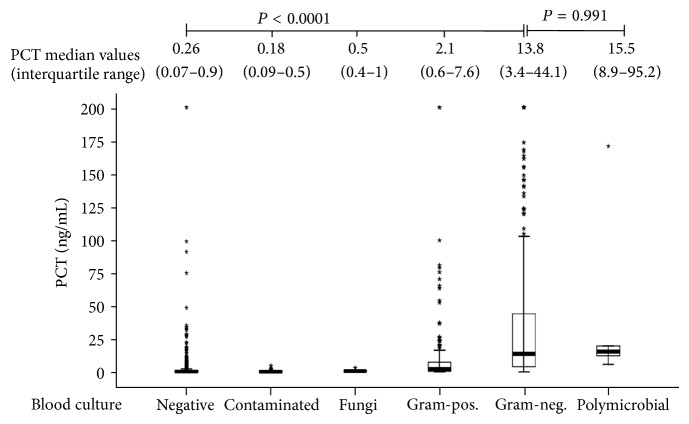
Comparison of PCT median values according to BC result.

PCT median values according to BC results are shown in Figure 1. Statistical analysis demonstrated that PCT median value corresponding to BCs positive for Gram-negative pathogens was significantly higher than those corresponding to negative or contaminated BCs and to BCs positive for fungal or Gram-positive pathogens, but not to polymicrobial BCs (Figure 1).

**Figure 2:**
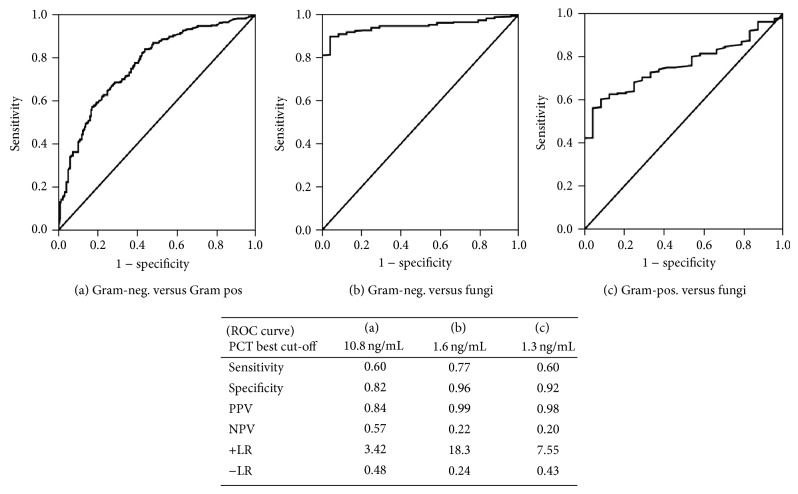
Receiver operating characteristic (ROC) curves of different cut-offs of PCT in differentiating: (a) Gram-negative bacteria from Gram-positive bacteria (AUC 0.765, 95% CI 0.725–0.805; *P* < 0.0001); (b) Gram-negative bacteria from fungi (AUC 0.944, 95% CI 0.919–0.969, *P* < 0.0001); (c) Gram-positive bacteria from fungi (AUC 0.763, 95% CI 0.693–0.832; *P* < 0.0001). Sensitivity, Specificity, Positive Predictive Value (PPV), Negative Predictive Value (NPV), Positive Likelihood Ratio (+LR), and Negative Likelihood Ratio (−LR) are reported for the best cut-off values found in each ROC.

To evaluate the PCT diagnostic accuracy in predicting causative organisms of bloodstream infections, ROC analysis was performed in monomicrobial BCs (Figure 2). The best diagnostic accuracy in discriminating Gram-negative from Gram-positive infections was at the cut-off value of 10.8 ng/mL, Gram-negative from fungal infections at 1.6 ng/mL, and Gram-positive from fungal infections at 1.3 ng/mL. The best values were found in discriminating Gram-negative or Gram-positive bacteria from fungi (Figure 2).

**Figure 3:**
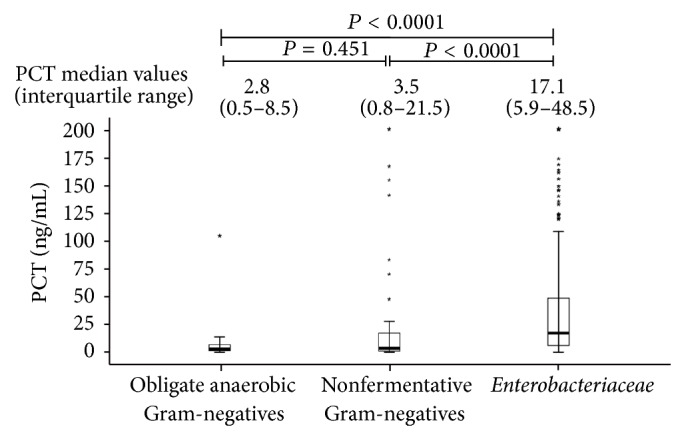
Comparison of PCT median values in bloodstream infections by* Enterobacteriaceae*, non-fermentative Gram-negative bacteria, or obligate anaerobic Gram-negative bacteria.

**Figure 4:**
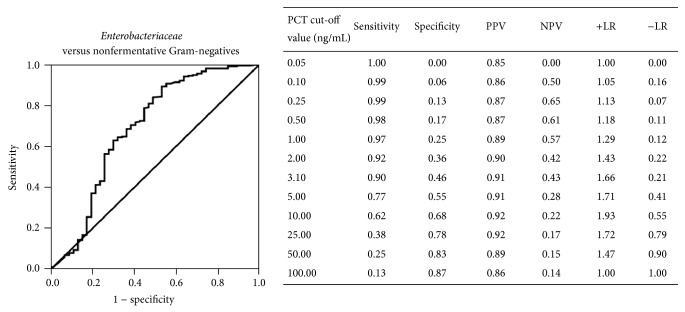
Receiver operating characteristic (ROC) curve of different cut-offs of PCT in differentiating* Enterobacteriaceae* from nonfermentative Gram-negative bacteria (AUC 0.691, 95% CI 0.593–0.789, *P* < 0.0001). Sensitivity, specificity, positive predictive value (PPV), negative predictive value (NPV), positive likelihood ratio (+LR), and negative likelihood ratio (−LR) of different cut-off values are reported.

Table 2 shows median PCT values corresponding to the different microbial species isolated in two or more patients with monomicrobial bacteremias, and Table 3 reports PCT values corresponding to pathogens isolated only in one patient or to polymicrobial infections. To evaluate the possibility that different PCT values could correspond to different microbial groups, PCT median values obtained in monomicrobial bloodstream infections by different species were compared. Among Gram-positives, median values found for* Streptococcus pneumoniae* or* Staphylococcus aureus* were significantly higher than those found for enterococci (0.8 ng/mL, IQR 0.4–2.3, and *P* = 0.001) or streptococci other than* S*.* pneumoniae* (1.4 ng/mL, IQR 0.3–3.9, and *P* = 0.005). No significant difference was found among different yeast species (data not shown). In bloodstream infections by Gram-negatives, PCT median value corresponding to* Enterobacteriaceae* (17.1 ng/mL, IQR 5.9–48.5) was significantly higher than that found for nonfermentative (3.5 ng/mL, IQR 0.8–21.5, and *P* < 0.0001) or obligate anaerobic bacteria (2.8 ng/mL, IQR 0.5–8.5, and *P* < 0.0001) (Figure 3). ROC analysis showed that the best cut-off for PCT in discriminating* Enterobacteriaceae* from nonfermentative Gram-negative bacteria was 3.1 ng/mL, with 90% sensitivity and 91% PPV (Figure 4).

## 4. Discussion

The main findings of this study are that, in patients with suspected sepsis, the PCT cut-off value of 10.8 ng/mL could be of help in predicting an infection caused by Gram-negatives, with a specificity of 82.5%. A cut-off of 3.1 ng/mL could be of help in excluding an infection caused by* Enterobacteriaceae* but not by nonfermentative Gram-negatives, with a sensitivity of 90.1%. These results suggest that PCT could be of some help to clinicians in evaluating the more appropriate initial antimicrobial therapy in the cases in which, even if informed of the presence of Gram-negative bacilli in patients' BC, they have to wait further 24–48 hours for species identification. This could be a relevant issue given that, in bloodstream infections by antibiotic-resistant nonfermentative Gram-negatives, such as* Pseudomonas aeruginosa*, an inappropriate initial antimicrobial therapy is strongly associated with adverse outcome [11, 12].

The ability of PCT to discriminate infections by Gram-positive or Gram-negative organisms has been recently described. Charles et al. [14], in a retrospective study on 97 bacteremia episodes, found that serum PCT levels were markedly greater for Gram-negatives than for Gram-positives, with an AUC of 0.79. Similarly, Koivula et al. [15] showed that elevated levels of PCT within 24 hours after the onset of fever predict Gram-negative bacteremia in hematological patients. Brodská et al. [13] in a retrospective study evaluating 166 patients found that PCT cut-off of 15 ng/mL can discriminate between sepses caused by Gram-negative bacteria or by Gram-positives and fungi, with a specificity of 87.8%. The different cut-off found in this study may be due to its greater sample size, with fourfold* Acinetobacter baumannii* and nearly threefold* Pseudomonas aeruginosa* isolates, associated with lower median PCT values, and to its prospective nature, limiting possible selection bias.

Although the mechanism underlying different PCT production in response to different bacterial pathogens is not completely clear, it could possibly be explained by the different interaction of Gram-positive or Gram-negative bacteria with host's cells, involving lipoteichoic acids or LPS, respectively, and different pathogen-associated molecular patterns (PAMPs), engaging different TLRs, expressed on human cells [7]. In particular, Gram-positive bacteria activate the TLR2 pathway [20, 21], whereas Gram-negative bacteria the TLR4 pathway [22], resulting in different production of inflammatory cytokines, such as interleukin-1*β*, interleukin-6 (IL-6), and tumor necrosis factor-*α*, that ultimately stimulate ubiquitous transcription of calcitonin-mRNA and release of PCT from multiple tissues throughout the body [1, 23].

To the best of our knowledge, this is the first study showing a significant difference in the PCT values between bloodstream infections sustained by* Enterobacteriaceae* and those caused by nonfermentative Gram-negatives. Interestingly, ROC analysis suggests that although PCT values >3.1 ng/mL do not discriminate between the two groups of pathogens, values ≤3.1 ng/mL are indicative of a low probability of bloodstream infection by* Enterobacteriaceae*. These results are in line with the findings of Elson et al. [24], demonstrating that* Enterobacteriaceae* such as* Escherichia coli* and* Klebsiella pneumoniae*, at a concentration of 10^4^ cells/mL, induced a greater in vitro IL-6 production by human umbilical vein endothelial cells than* P. aeruginosa* that, even at a concentrations of 10^6^ cells/mL, produced low levels of IL-6, a known inducer of PCT [23].

It is conceivable that the high median PCT values found in polymicrobial bloodstream infections could be attributable to the presence of Gram-negative bacteria in all of them, specifically,* Enterobacteriaceae* in three out of five cases, but this issue needs to be verified with further studies.

We found that PCT optimally discriminated between Gram-negative and fungal infections at the best cut-off of 1.6 ng/mL and, though with less accuracy, between Gram-positive and fungal infections. Similarly, Martini et al. [25] in 48 critically ill surgical patients at high risk for fungal infection with signs of sepsis found that PCT cut-off of 2.0 ng/mL can discriminate between* Candida* and bacterial sepsis. Conversely, Fu et al. [26], in a population of 85 critically ill patients, found a cut-off of 8.06 ng/mL in discriminating between candidemia and Gram-negative bacterial sepsis. These differences highlight how the results can greatly depend on the type of the studied patients population, as PCT values can significantly differ in different clinical settings [27]. Indeed, the present study was carried out in a large population of 1,949 patients mainly from internal medicine wards, for which blood cultures were collected together with sera for PCT determination. These inclusion criteria could have selected patients with high suspicion of sepsis, as evidenced by the high pathogen detection rate (30.3%) found.

This study has some limitations. First, the discriminatory power found for PCT could have been confounded by the lack of patients' baseline characteristics and comorbidities. Indeed, information about factors that can influence PCT levels, such as recent transplantation, severe and prolonged cardiogenic shock, heat shock, severe pancreatitis, rhabdomyolysis, autoimmune disorders, and others [28], was not available for all the patients. Second, since the study has been conducted in a wide range of patients, the results are not specifically applicable to selected settings. Third, as intervals between the onset of symptoms and sampling were not available, it was not possible to rule out that some low PCT values could have been due to early sampling, given that PCT increases during the first six hours of infection [29, 30]. Finally, the low number of bacteremias from rarely encountered pathogens does not allow any conclusion about the significance of PCT in these infections (Table 3). Nevertheless, the large cohort studied, the systematic approach to PCT measurement, the fact that all the bloodstream infections included in the study were microbiologically documented, and that the spectrum of causative organisms was consistent with a large prospective multicenter Italian study, with* E. coli* and* S. aureus* as the most frequent pathogens [31], could have strengthened the results of this study.

## 5. Conclusions

In conclusion, PCT may be of value to distinguish Gram-negative from Gram-positive and fungal infections; nevertheless, its utility to predict different microorganisms needs to be assessed in further studies including detailed patient information. The findings of the present study show that PCT cut-off of ≥10.8 ng/mL could suggest an infection by Gram-negatives, and the cut-off ≤3.1 ng/mL could suggest exclusion of infection by Enterobacteriaceae. A PCT cut-off >1.3 ng/mL could be of help in ruling out a fungal bloodstream infection.

## Figures and Tables

**Table 1:** Demographic and clinical characteristics of the 1,949 patients included in the study.

Variable	Values
Males	1,150 (59%)
Females	799 (41%)
Age (years)	74 (IQR 62–83)^*^
Ward of hospitalization	
Medical	1,735 (89%)
Surgical	179 (9.2%)
Intensive Care Unit	35 (1.8%)
Antimicrobial therapy before sampling	1,553 (79.7%)
Blood culture	
Negative	1,286 (66%)
Contaminated	72 (3.7%)
Monomicrobial	586 (30.6%)
Polymicrobial	5 (0.3%)

^*^Median value and interquartile range (IQR).

**Table 2:** PCT median values corresponding to pathogens that were isolated from two or more patients with monomicrobial bloodstream infections.

Pathogen	Number of patients	Median PCT values (interquartile range) (ng/mL)
Gram-positives		
*Staphylococcus aureus *	103	3.6 (1.3–9.3)
*Enterococcus faecalis *	43	0.5 (0.3–2.2)
*Enterococcus faecium *	18	1.6 (0.9–2)
*Streptococcus pneumoniae *	18	6.9 (3.3–23.9)
*Streptococcus pyogenes *	5	2.1 (0.2–2.2)
*Streptococcus gallolyticus *	4	14 (1.6–26.1)
*Listeria monocytogenes *	3	1.1 (0.6–1.2)
*Streptococcus parasanguinis *	3	0.3 (0.2–2.7)
*Streptococcus agalactiae *	3	1.8 (1–7.9)
*Streptococcus mutans *	2	6.3 (0.6–12.1)
*Streptococcus bovis *	2	1.1 (0.7–1.4)
*Streptococcus sanguinis *	2	5.4 (0.1–10.7)
*Propionibacterium acnes *	2	0.07 (0.05–0.1)
Gram-negatives		
*Enterobacteriaceae *		
*Escherichia coli *	183	18.5 (6.5–56.4)
*Klebsiella pneumoniae *	56	22.3 (9.6–52.4)
*Enterobacter cloacae *	14	5.5 (3.5–7.5)
*Proteus mirabilis *	12	11.3 (8.3–16.5)
*Serratia marcescens *	5	14.9 (3.8–15.5)
*Klebsiella oxytoca *	5	2.3 (1.1–3.7)
*Salmonella typhi *	3	23.2 (18–24.7)
*Pantoea agglomerans *	3	21.5 (12.5–110.7)
*Enterobacter aerogenes *	2	71.8 (68.2–75.4)
*Citrobacter koseri *	2	21 (14.4–27.7)
Nonfermentative obligate aerobic		
*Pseudomonas aeruginosa *	21	6.8 (1.3–11.9)
*Acinetobacter baumannii *	16	2.2 (0.6–7.4)
*Stenotrophomonas maltophilia *	4	20.5 (9.4–166.6)
Obligate anaerobic		
*Bacteroides fragilis *	8	2.8 (0.5–8.5)
Fungi		
*Candida albicans *	12	0.5 (0.3–1.2)
*Candida parapsilosis *	3	0.6 (0.5–0.9)
*Candida lusitaniae *	5	0.6 (0.4–0.7)

**Table 3:** PCT values corresponding to pathogens isolated from single patients with monomicrobial or polymicrobial bloodstream infections.

Bloodstream infection from	Pathogen	PCT values (ng/mL)
Gram-positives	*Abiotrophia defectiva *	1.31
	*Capnocytophaga canimorsus *	0.87
	*Capnocytophaga sputigena *	6.63
	*Clostridium paraputrificum *	4.52
	*Enterococcus avium *	3.54
	*Kytococcus sedentarius *	0.40
	*Peptostreptococcus *spp.	0.25
	*Streptococcus anginosus *	0.21
	*Streptococcus gordonii *	0.45

Gram-negatives	*Acinetobacter junii *	1.70
	*Bacteroides thetaiotaomicron *	8.49
	*Burkholderia gladioli *	0.42
	*Fusobacterium necrophorum *	2.24
	*Fusobacterium nucleatum *	0.38
	*Haemophilus influenzae *	0.48
	*Moraxella catarrhalis *	0.44
	*Moraxella nonliquefaciens *	0.98
	*Neisseria meningitidis *	24.17
	*Providencia rettgeri *	0.90
	*Pseudomonas putida *	2.49

Fungi	*Candida glabrata *	0.66
	*Candida krusei *	0.72
	*Candida pelliculosa *	1.06
	*Candida tropicalis *	0.13

Polymicrobial	*Enterococcus faecalis* and *Serratia marcescens *	15.5
	*Enterococcus faecalis* and *Stenotrophomonas maltophilia *	12.3
	*Enterococcus faecium* and *Klebsiella pneumoniae *	19.5
	*Staphylococcus aureus* and *Acinetobacter baumannii *	5.61
	*Staphylococcus aureus* and *Proteus mirabilis *	170.9
